# Exploring mechanisms of Chaihu-Shugan-San against liver fibrosis by integrated multi-omics and network pharmacology approach

**DOI:** 10.1042/BSR20221030

**Published:** 2022-07-20

**Authors:** Zhihao Xie, Zhiying Xie, Nicolas Pineda Trujillo, Ting Yang, Chunxia Yang

**Affiliations:** 1 Department of Epidemiology and Biostatistics, West China School of Public Health and West China Fourth Hospital, Sichuan University, Chengdu 610041, China; 2 West China-PUMC C.C. Chen Institute of Health, Sichuan University, Chengdu 610041, China; 3 Department of Neurology, Peking University First Hospital, Beijing 100034, China; 4 Grupo Mapeo Genetico, Departamento de Pediatría, Facultad de Medicina, Universidad de Antioquia, Medellín 050010470, Colombia; 5 School of Public Health, Guizhou Medical University, Guiyang 550025, China

**Keywords:** Chaihu-Shugan-San, computational biology, liver fibrosis, network pharmacology, prognosis

## Abstract

Chaihu-Shugan-San (CHSGS), a noted traditional Chinese medicine formula, has been used as a complementary and alternative therapy for liver fibrosis. However, the antifibrotic mechanisms of CHSGS still remain unclear. Thus, we used network pharmacology approach in combination with single cell and bulk transcriptomics to elucidate the antifibrotic mechanisms of CHSGS. We first screened out 134 bioactive ingredients of CHSGS through the defined criteria. Then, 1150 genes were predicted to be targets for CHSGS, while 625 liver fibrosis-associated genes were identified by single cell transcriptomics analysis. Next, 71 intersecting genes of CHSGS and liver fibrosis were defined as the therapeutic targets in CHSGS against liver fibrosis. Further, 21 core targets and 12 core ingredients of CHSGS against liver fibrosis were also identified. Meanwhile, enrichment analyses of core targets highlighted that the key mechanisms of CHSGS against liver fibrosis include modulation of inflammation responses, inhibition of angiogenesis, and regulation of ECM remodeling, of which the most important mechanism was the regulation of ECM remodeling. The molecular docking simulation validated strong binding affinity between the core targets and core ingredients. Furthermore, 62-gene signature may be used for determining the prognosis in cirrhotic patients based on the results of ssGSEA-Cox analysis. In conclusion, the present study revealed the multiple pharmacological targets and therapeutic mechanisms of CHSGS against liver fibrosis, which may thus serve as an effective antifibrotic therapy. Meanwhile, CHSGS may improve survival of patients with liver cirrhosis by the interaction of 62-gene signature.

## Introduction

Liver fibrosis is a fibrous scar formed by the excessive deposition of extracellular matrix (ECM) proteins, which mainly includes collagens type I and type III [1,2]. It can be classified into four stages of increasing severity. Stages 3 and 4 of liver fibrosis are classified as liver cirrhosis [3]. Due to lack of effective antifibrotic therapies, liver fibrosis progression eventually leads to cirrhosis. Liver cirrhosis is a world health challenge with estimates indicating that 106 million people have decompensated cirrhosis and 112 million people have compensated cirrhosis globally [4]. In 2017, liver cirrhosis caused over 1.3 million deaths globally, which accounted for 2.4% of global deaths [4]. Present western therapies for liver fibrosis are confined to etiological therapies, aiming at removal of the various primary causes of liver fibrosis. Several types of single mechanism agents have been designed for antifibrotic therapies, based on the protection of hepatocytes, suppression of fibrogenesis, and promotion of fibrosis resolution [2,5,6]. However, there are currently no approved western drugs for the treatment of liver fibrosis [2,7]. Thus, the broad range of bioactivities of natural herbs has raised the interest in their possible antifibrosis effects [8].

Traditional Chinese medicine (TCM), as one of the common natural herbal medicines, has been widely applied for treating liver diseases in China. TCM formula, comprised of multiple components, may play a therapeutic role in liver fibrosis via multitargets, multiple signal pathways and mechanisms [9–11]. Chaihu-Shugan-San (CHSGS) agent consists of seven Chinese herbs: *Bupleurum chinense* DC. (*Radix Bupleuri*, Chaihu, CH), *Citrus aurantium* L. (*Aurantii Fructus*, Zhiqiao, ZQ), Ligusticum striatum DC. (*Chuanxiong Rhizoma*, Chuanxiong, CX), *Citrus reticulata* Blanco (*Citrus Reticulata*, Chenpi, CP), *Cyperus rotundus* L. (*Cyperi Rhizoma*, Xiangfu, XF), *Paeonia lactiflora* Pall. (*Paeoniae Radix Alba*, Baishao, BS), and *Glycyrrhiza uralensis* Fisch. (*Radix Glycyrrhrizae Urelensis*, Gancao, GC). It has been widely used as a common TCM formula for the treatment of liver diseases in China. CHSGS can alleviate chronic inflammation in nonalcoholic fatty liver rats through the NLRP3 inflammasome pathway [12]. In addition, the p38 MAPK pathway is also the effective target for CHSGS against nonalcoholic steatohepatitis [13]. Further, a retrospective study revealed that CHSGS was the most efficient TCM formula that improved the survival rate for liver cancer [14]. Moreover, our previous study also showed that patients with liver cirrhosis received CHSGS had a significantly lower death risk than those without TCM use [15]. It could be inferred that a sustained CHSGS therapy of cirrhosis might induce regression of fibrosis, which lead to a longer survival time of patients with liver cirrhosis. However, the bioactive ingredients, pharmacological targets, and mechanisms of CHSGS against liver fibrosis still remain unclear and need to be elucidated in detail.

Conventional experimental methods cannot well describe the complex pharmacological mechanisms of TCM. Furthermore, in the absence of knowledge on specific mechanisms, the application of TCM is not widespread globally [16]. Due to the rapid development of bioinformatics in recent years, network pharmacology analysis as a new tool that can help elucidate complex mechanisms of TCM in treating complex diseases. In the present study, we used network pharmacology approach in combination with single cell and bulk transcriptomics to elucidate the antifibrotic mechanisms of CHSGS. Liver nonparenchymal cells (NPCs), including immune cells, endothelial cells, cholangiocytes, hepatic stellate cells (HSCs) and Kupffer cells, which facilitate the diverse functions of hepatocyte and have complex interactions in liver fibrosis [7,17]. Application of single-cell RNA sequencing (scRNA-seq) for gene expression analysis has been conducted on NPCs to study the mechanisms of human liver fibrosis [7,17,18]. Herein, we integrated two public scRNA-seq data of NPCs derived from cirrhotic and healthy human livers to determine the associated genes of liver fibrosis. Further, network visualization was used to elaborate the detailed therapeutic mechanisms of CHSGS against liver fibrosis.

## Materials and methods

### Study design

**Figure 1:**
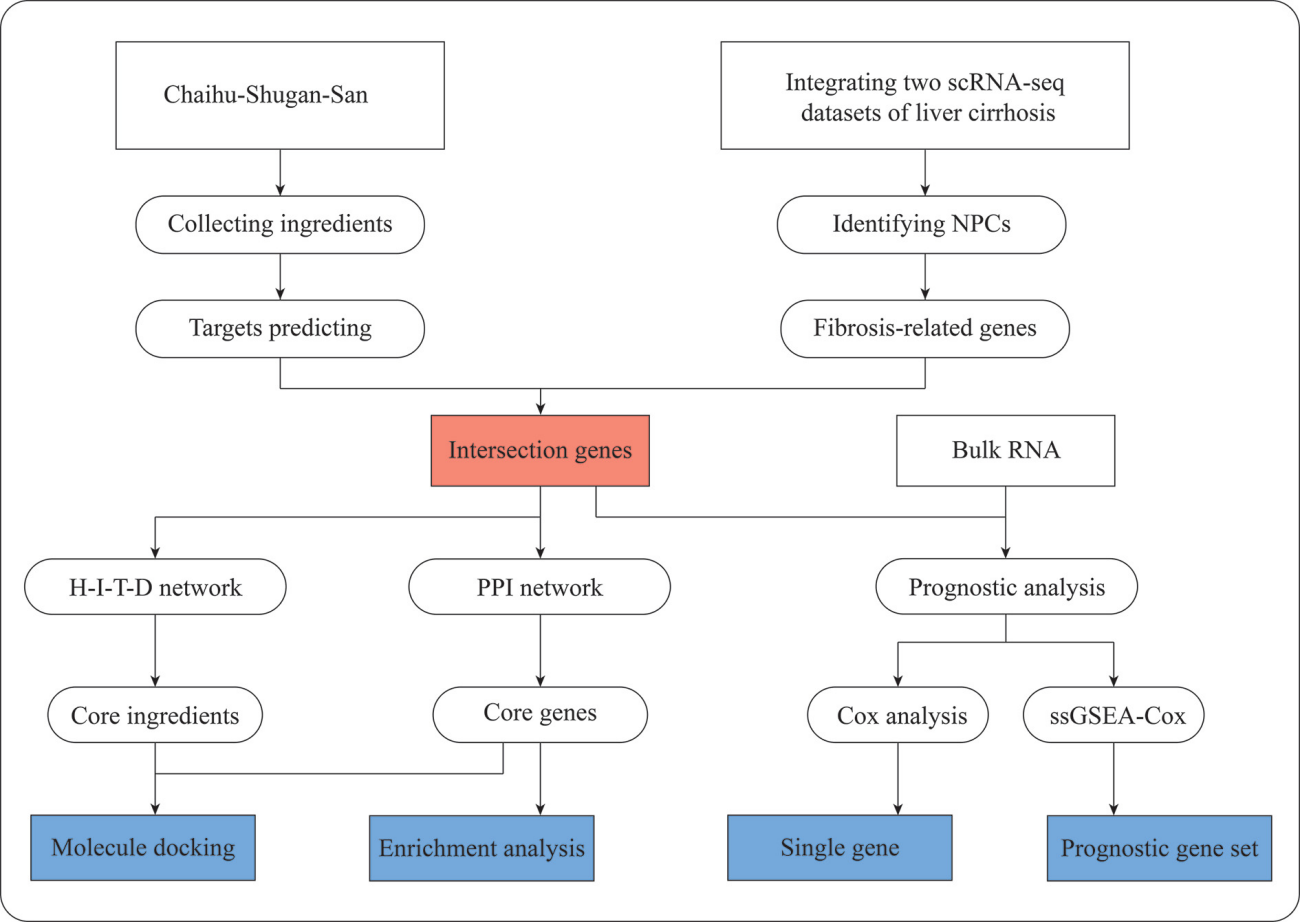
Flowchart of the analysis of the mechanisms of Chaihu-Shugan-San on antifibrosis and improving survival of liver cirrhosis Abbreviations: H-I-T-D, herb-ingredient-target-disease; NPC, nonparenchymal cell; PPI, protein–protein interaction.

In the present study, liver fibrosis-associated genes were identified using the differential gene expression (DEG) analysis with scRNA-seq data of liver NPCs. The pharmacological targets for CHSGS were obtained from web tools, including TCMSP [19], SEA [20], and SwissTargetPrediction [21]. Then, the intersection genes of CHSGS and liver fibrosis were used to construct protein–protein interaction (PPI) network. Meanwhile, using Cox and single-sample gene set enrichment analysis combined with Cox regression algorithm (ssGSEA-Cox) methods with the public bulk transcriptomics, we evaluated whether the intersection genes were related to the survival of patients with liver cirrhosis. Further, enrichment analyses were utilized to reveal the therapeutic mechanisms of CHSGS on antifibrosis. Moreover, molecular docking was applied for analyzing the binding capacity of core ingredients and core target proteins in CHSGS against liver fibrosis (Figure 1).

### Single-cell RNA sequencing data analysis and identifying liver fibrosis-related genes

To identify the target genes associated with liver fibrosis, we integrated the public scRNA-seq data of liver NPCs from the GEO database [GSE136103], which includes five cirrhotic and five healthy human livers [17], and the ArrayExpress database [E-MTAB-10553], which includes six healthy human livers [7]. All scRNA-seq data preprocessing and analyzing were performed with the R package Seurat (Version 4.0.4). The initial integrated dataset contained 77,750 NPCs, of whom, 62,210 cells obtained from [GSE136103] for 33,538 genes and 15,540 cells obtained from [E-MTAB-10553] for 23,396 genes. Genes expressed in at least ten cells were kept, as were cells that expressed at least 200 genes or with a mitochondrial gene percentage of <30%. Using the Seurat function *FindVariableFeatures* to identify 2000 highly variable genes in the log-normalized RNA counts. Then, function *IntegrateData* was used to merge the two scRNA-seq datasets. After processing the above step, a total of 76,467 cells were retained for downstream analysis.

Next, we conducted principal component analysis using the integrated variable genes for dimension reduction and estimated significant principal components based on the Seurat function *ElbowPlot*. The first 12 principal components were opted as input for shared nearest neighbour graph-based clustering; the clustering resolution was set to 0.4 based on the function *clustree*. With the above parameters, 76,467 cells were classified into 17 initial clusters using the Seurat function *FindClusters*. Further, the cells clustering results visualization were constructed using uniform manifold approximation and projection with the same 12 principal components as the related clustering. In order to identify the genes associated with liver fibrosis, DEG analysis was performed in the Seurat function *FindMarkers* using Wilcoxon rank sum test. Only genes expressed in at least 25% of the compared cells and with a log fold-change of at least 0.7 were retained.

### Collecting chemical ingredients of CHSGS

First, we extracted all chemical ingredients of CHSGS from the TCMSP [19], TCMID [22], and SymMap [23] databases. Then, absorption-distribution-metabolism-excretion (ADME) models, including oral bioavailability (OB) >30%, gastrointestinal (GI) absorption of high, druglikeness (DL) >0.18, and Lipinski’s rules were used to screen the bioactive ingredients of CHSGS. The details of Lipinski’s rules [24] were molecular weight ≤500 g/mol, number of donor hydrogen bonds (Hdon) ≤5, number of acceptor hydrogen bonds (Hacc) ≤10, and implicit log *P* method (iLOGP) ≤5 [25].

### Acquisition of CHSGS-pharmacological targets

The initial pharmacological targets of CHSGS were obtained from TCMSP [19] Database. We then utilized the function *obabel* setting from Open Babel v2.4.1 to convert canonical SMILES of the bioactive ingredients for targets prediction. Next, the SEA [20] and SwissTargetPrediction [21] online tools were also used to predict the targets of bioactive ingredients in CHSGS.

### Determining core targets and ingredients of CHSGS against fibrosis

The overlapping target genes of CHSGS against liver fibrosis, obtained from the intersection of CHSGS-pharmacological targets with liver fibrosis-related genes, were utilized to construct the PPI network in the STRING database [26]. Then, the Cytoscape software (Version 3.9.0) was applied for performing the PPI network visualization. Core target genes were identified using network centrality method. Specifically, the centralities of PPI network were first calculated by the CytoNCA tool setting from Cytoscape software, including betweenness, closeness, degree, eigenvector, local average connectivity-based method, network, subgraph, and information. The overlapping targets with all centrality parameters greater than the median centralities of the PPI network were then defined as core targets. Next, we used Cytoscape software to establish the networks of ingredient-target and herb-ingredient-target-disease. Similarly, we also used the median centrality parameters of the herb-ingredient-target-disease network to determine the core ingredients of CHSGS against liver fibrosis.

### Enrichment analysis for the core targets

R packages, including ClusterProfiler (Version 4.2.0), org.Hs.eg.Db (3.14.0) and ReactomePA (Version 1.38.0), were applied to perform Gene Ontology (GO) and Reactome pathways enrichment analyses of the core target genes of CHSGS against liver fibrosis. The cutoff criteria of GO and Reactome pathways enrichment analyses were adjusted *P*<0.01 with *q-*value < 0.05, adjusted *P*<0.05 with *q-*value < 0.05, respectively.

### Identification of the prognostic gene signature

To evaluate whether the intersection genes of CHSGS and liver fibrosis were associated with the survival of liver cirrhosis patients, we used a bulk RNA-seq dataset from GEO database [GSE15654] with 216 liver cirrhosis patients [27]. The survival analysis of single intersection gene was conducted using Cox proportional hazards regression with a hazard ratio (HR). Moreover, we constructed a single-sample gene set enrichment analysis [28] combined with Cox regression algorithm (ssGSEA-Cox) to find the optimal prognostic gene signature of CHSGS against liver cirrhosis. The ssGSEA-Cox method consists of three main steps. First, calculating enrichment scores of the given gene set in each sample through ssGSEA method. Second, the samples were divided into two subgroups (high- and low-enrichment scores subgroups) based on the median value of the enrichment scores; the univariate Cox analysis was then performed to determine whether the enrichment scores were significantly associated with death in liver cirrhosis. Last, using combinational algorithm to seek the optimal gene set, which was with maximal numbers of genes and to be significantly associated with survival of liver cirrhosis patients.

### Molecular docking of target proteins and molecules

Molecular docking was applied for analyzing the binding and interaction situation of core ingredients and core target proteins in CHSGS against liver fibrosis. The three-dimensional (3D) structure of the core target proteins and molecular structure of the core ingredients were obtained from the PDB (https://www.rcsb.org/) database and PubChem (https://pubchem.ncbi.nlm.nih.gov/) database, respectively. The initial preprocessing of removing water and original ligands of the core target proteins were performed by PyMol (Version 2.2.0) software. We then used MGLTools (Version 1.5.6) of Autodock software to add hydrogen and get PDBQT format files of the core macromolecules and ligands for further docking. Meanwhile, the docking active center parameters, wrapped the macromolecules, were set by the grid box function in the MGLTools. Finally, virtual docking was performed with the above parameters by using Autodock Vina (Version1.1.2) software [29] and the docking results were displayed by Discovery Studio Visualizer v21.1.0.

## Results

### Analysis of scRNA-seq data and liver fibrosis-associated genes

**Figure 2:**
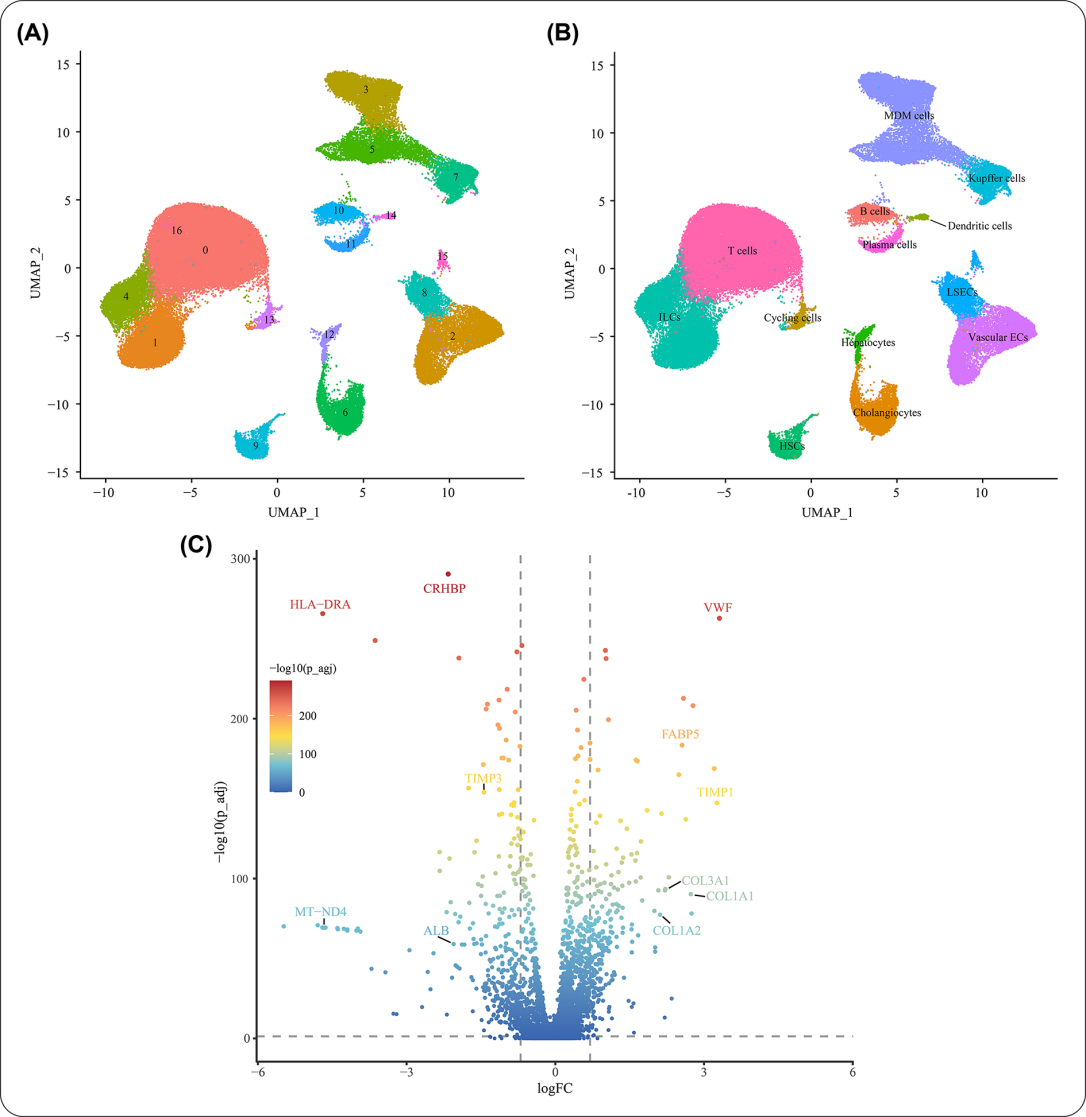
Analysis of scRNA-seq data for human liver NPCs (**A**) Initial clustering of the two integrated scRNA-seq datasets. (**B**) Cell lineages inferred from expression of the known marker genes. (**C**) Volcano plot of differential gene expression analysis between cirrhotic and healthy NPCs. Abbreviations: HSC, hepatic stellate cell; LSEC, liver sinusoidal endothelial cell; MDM, monocyte-derived macrophage; NPC, nonparenchymal cell; VEC, vascular endothelial cell.

The integrated scRNA-seq dataset was classified into 17 initial cell clusters (Figure 2A). After annotating with the known marker genes of cell lineages in liver (Supplementary Table S1), the integrated cells were identified by 13 distinct cell lineages: hepatic stellate cells (HSCs), Kupffer cells, monocyte-derived macrophages (MDMs), liver sinusoidal endothelial cells (LSECs), vascular endothelial cells (VECs), cholangiocytes, hepatocytes, T cells, innate lymphoid and natural killer cells (ILCs), B cells, plasma cells, dendritic cells (DCs), and cycling cells (Figure 2B). Although the two public datasets were focused on the hepatic NPCs, a small cluster of hepatocytes were retained during the dissociation process. Since in our research we focused on the liver NPCs, including HSCs, Kupffer cells, MDMs, LESCs, VECs, cholangiocytes, and immune cells, the differential expressed genes of these cell lineages were identified as target genes of liver fibrosis. Finally, 625 liver fibrosis-associated genes were identified by DEG analysis, of which 390 were down-regulated and 235 were up-regulated (Figure 2C and Supplementary Table S2).

### Collection of the ingredients and targets in CHSGS

**Figure 3:**
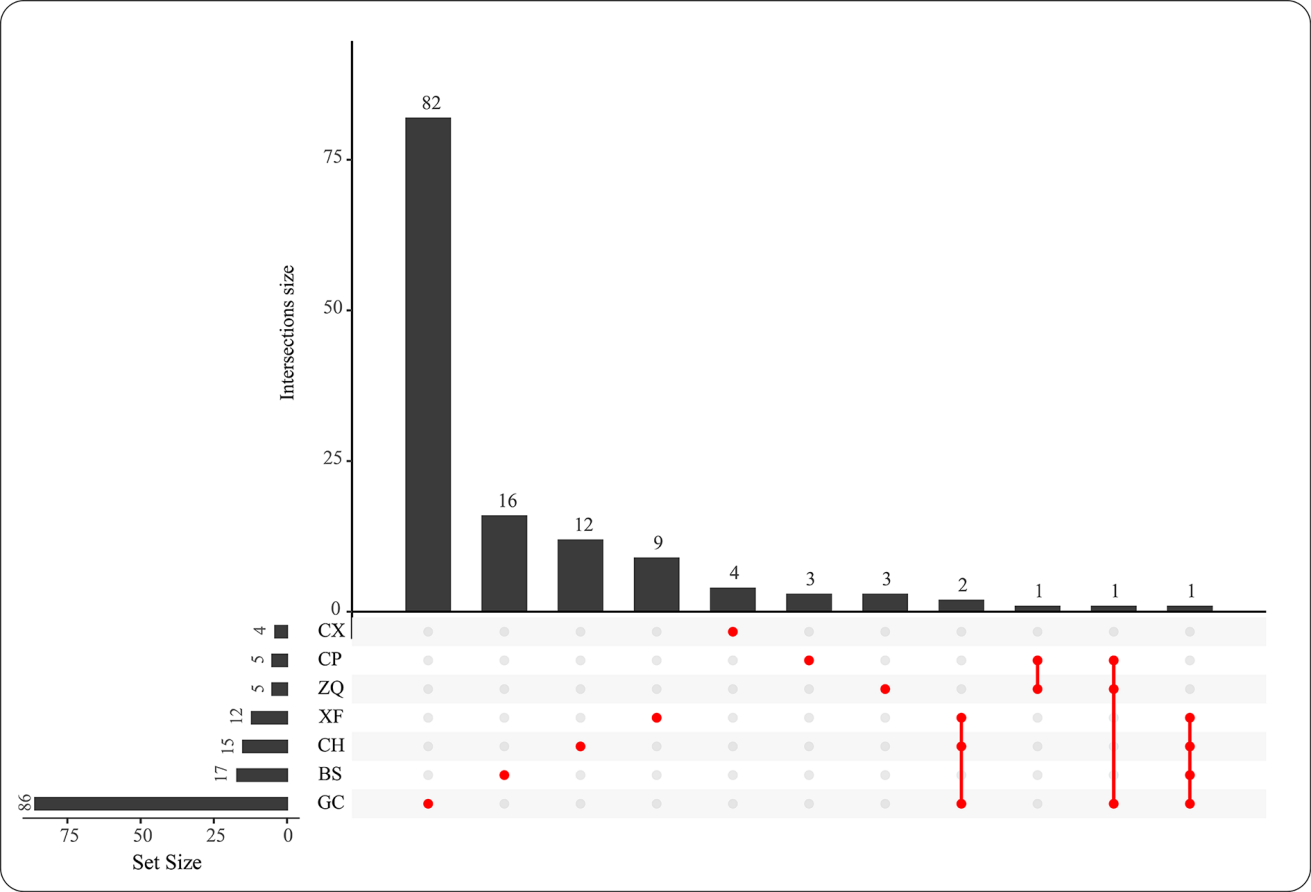
Specific and common ingredients of the seven herbs in Chaihu-Shugan-San

A total of 1075 chemical ingredients from seven Chinese herbs in CHSGS were obtained from TCMSP, TCMID, and SymMap databases, of which 134 ingredients passed the ADME screening (Supplementary Table S3). Among the 134 bioactive ingredients, five ingredients were shared by two or more Chinese herbs, including kaempferol (MOL000422), quercetin (MOL000098), isorhamnetin (MOL000354), naringenin (MOL004328), and nobiletin (MOL005828). In addition, CH, ZQ, CX, CP, XF, BS, and GC Chinese herbs had 12, 3, 4, 3, 9, 16, and 82 specific bioactive ingredients, respectively (Figure 3). It indicated that these multiple ingredients of CHSGS constitute a synergistic effect model to treat liver fibrosis.

**Figure 4:**
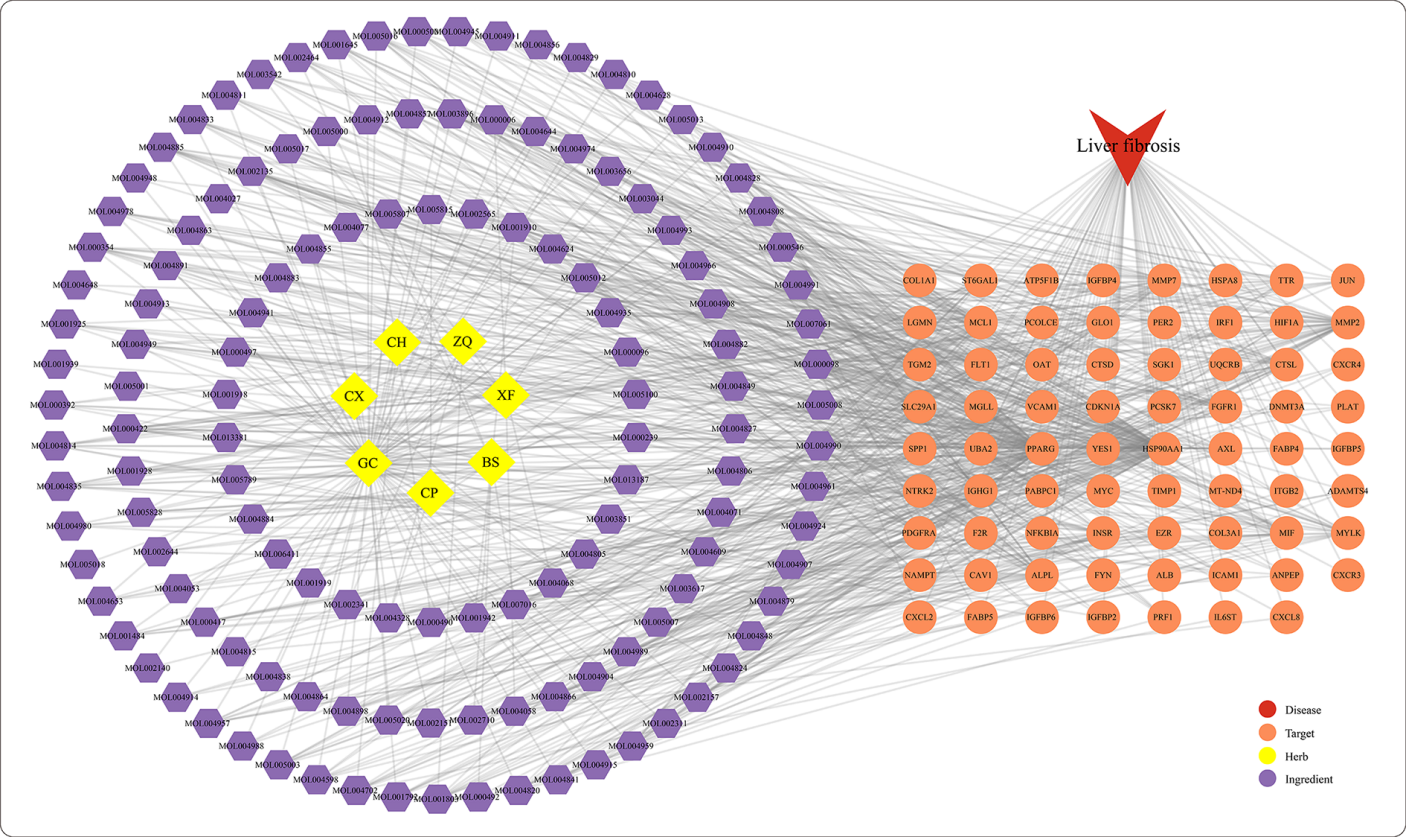
The herb-ingredient-target-disease network of Chaihu-Shugan-San against liver fibrosis

With the target predicting methods, a total of 1150 genes were predicted to be targets for CHSGS. Meanwhile, the ingredient-target network was established by Cytoscape software with 134 bioactive ingredients and 1150 target genes. Almost all bioactive ingredients had multiple targets that lead to a complex network with 10,854 interactions between ingredients and targets (Supplementary Table S4). In the ingredient-target network, there were 40 target genes per ingredients on average. Quercetin (MOL000098) had the majority of targets, with a frequency of 231. Further, the 71 intersection genes in CHSGS and liver fibrosis were used to construct the herb-ingredient-target-disease network, which was composed of 213 nodes and 848 edges (Figure 4). With the network centrality method of determining core ingredients, 12 core ingredients were identified, including quercetin (MOL000098), kaempferol (MOL000422), luteolin (MOL000006), isorhamnetin (MOL000354), 7-methoxy-2-methyl isoflavone (MOL003896), glyasperin B (MOL004808), kanzonols W (MOL004820), licoisoflavanone (MOL004885), quercetin der. (MOL004961), 7,2',4'-trihydroxy 5-methoxy-3 arylcoumarin (MOL004990), glyasperins M (MOL005007), and odoratin (MOL005016). These results suggested that CHSGS has characteristics of multiple ingredients and targets in the treatment of liver fibrosis.

### Identification and enrichment analysis of the targets of CHSGS against liver fibrosis

**Figure 5:**
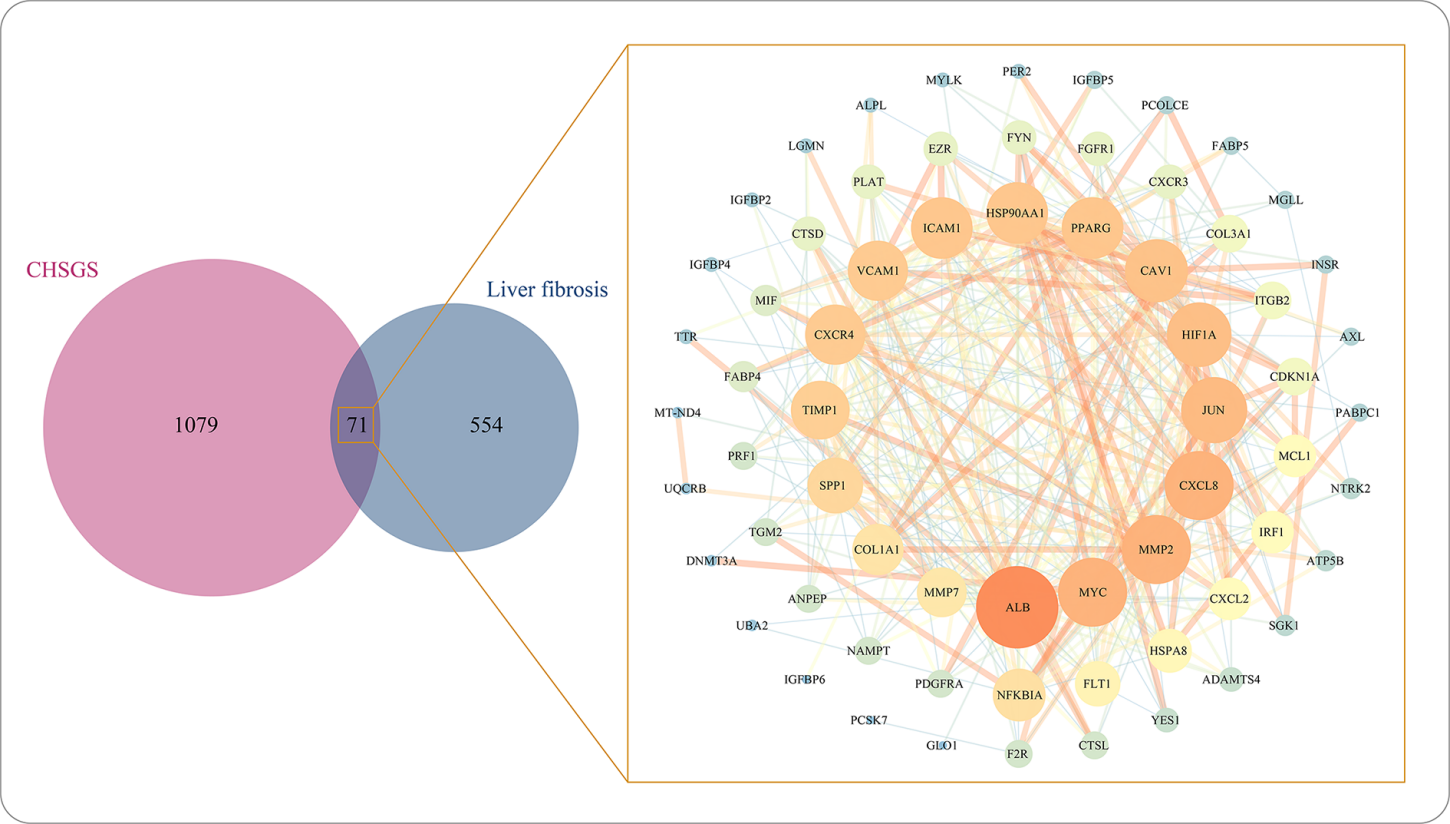
The protein–protein interaction network of 71 intersecting targets of Chaihu-Shugan-San against liver fibrosis

**Figure 6:**
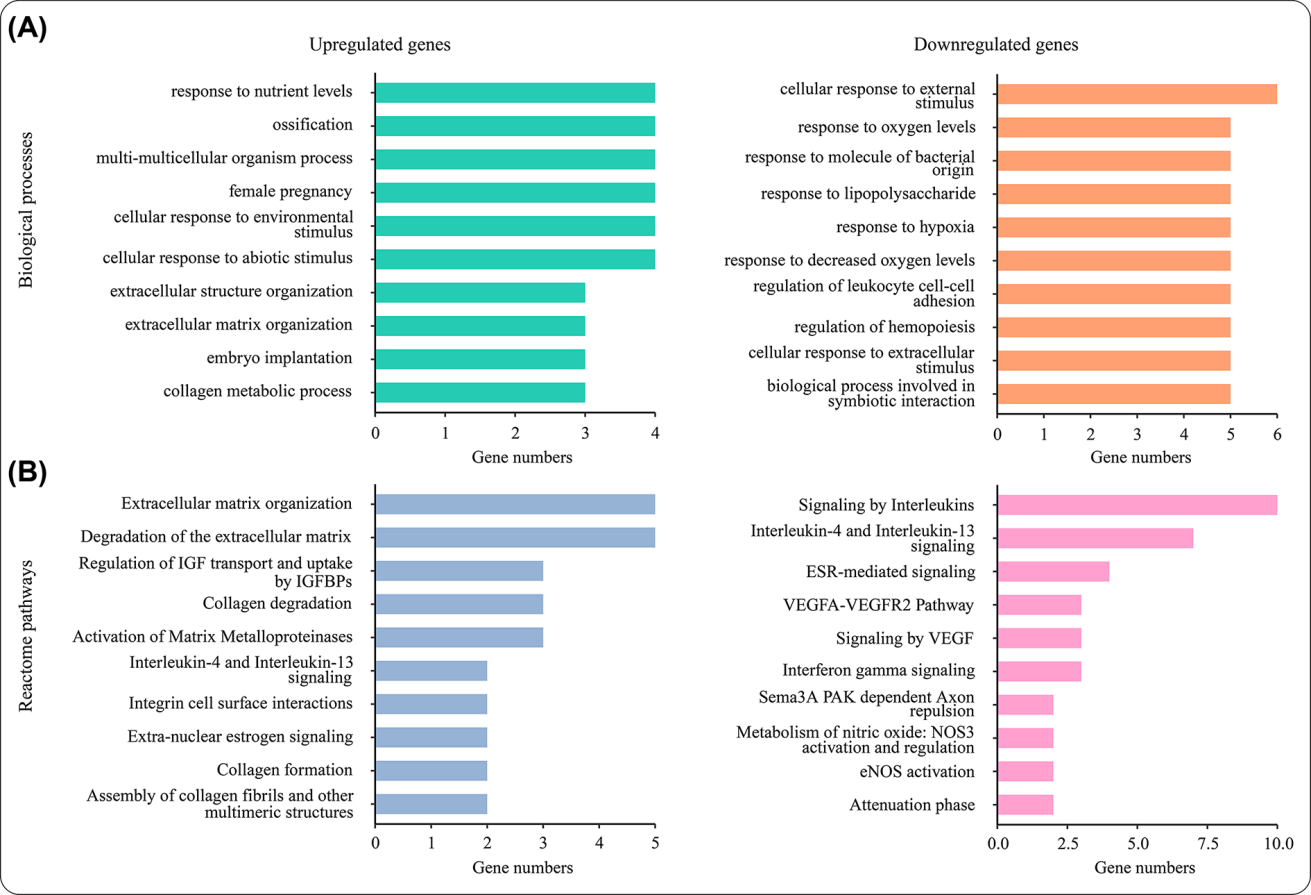
Enrichment analyses of core intersecting genes of Chaihu-Shugan-San against liver fibrosis (**A**) Biological process of core intersecting genes of Chaihu-Shugan-San against liver fibrosis. (**B**) Reactome pathway of core intersecting genes of CHSGS against liver fibrosis.

**Figure 7:**
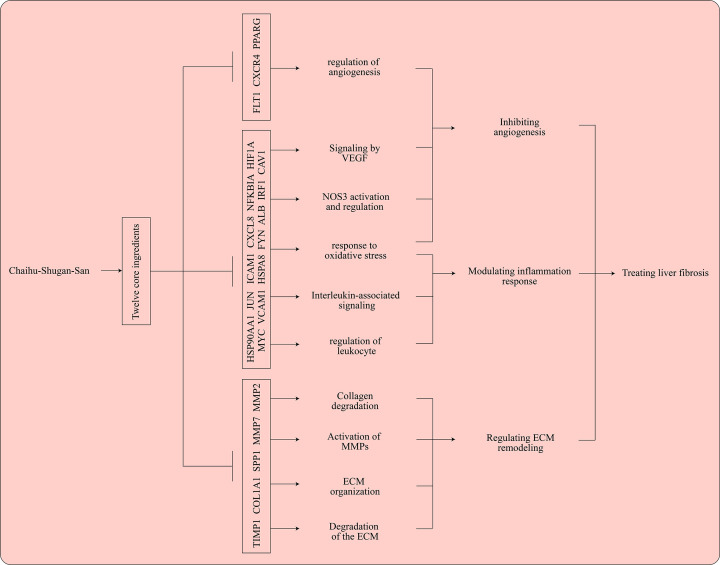
The specific mechanisms of Chaihu-Shugan-San against liver fibrosis ECM, extracellular matrix

In our research, 71 overlapping genes of CHSGS against liver fibrosis were identified by the intersection of 1150 CHSGS-pharmacological targets with 625 liver fibrosis-associated genes, which were queried in STRING online tool to construct the PPI network (Figure 5). After hiding the disconnected nodes in the PPI network, the nodes and edges were 66 and 365, respectively. With the network centrality method of identifying core genes, 21 core genes were identified, of which TIMP1, COL1A1, SPP1, FLT1, MMP7, CXCR4, PPARG and MMP2 genes were up-regulated and 13 genes including ALB, HSP90AA1, JUN, ICAM1, CXCL8, NFKBIA, HIF1A, MYC, VCAM1, HSPA8, FYN, CAV1, and IRF1 were down-regulated. These upregulated genes were involved in 46 biological processes (BPs) by using GO enrichment analysis, including cellular response to abiotic stimulus, cellular response to environmental stimulus, collagen metabolic process, ECM organization, and extracellular structure organization (Figure 6A and Supplementary Table S5). Besides, 16 pathways were found in the Reactome analysis of the up-regulated genes. Except interleukin-associated signal pathway, many of these pathways such as ECM organization, degradation of the ECM, collagen degradation, activation of matrix metalloproteinases, and assembly of collagen fibrils and other multimeric structures were involved in ECM remodeling, consequently resulting in liver fibrosis. (Figure 6B and Supplementary Table S6). In addition to up-regulated genes, down-regulated genes were related to inflammation response, oxidative stress, interleukin-associated signaling, NOS3 activation and regulation and VEGF signaling pathway (Figure 6A,B; Supplementary Tables S5 and S6). In summary, the key mechanisms of CHSGS against liver fibrosis were shown in Figure 7, including modulation of inflammation responses, inhibition of angiogenesis, and regulation of ECM remodeling. Among these key mechanisms, the regulation of ECM remodeling was identified as the most important mechanism of CHSGS against liver fibrosis.

### Intersection genes as prognostic gene signature of liver cirrhosis

**Figure 8:**
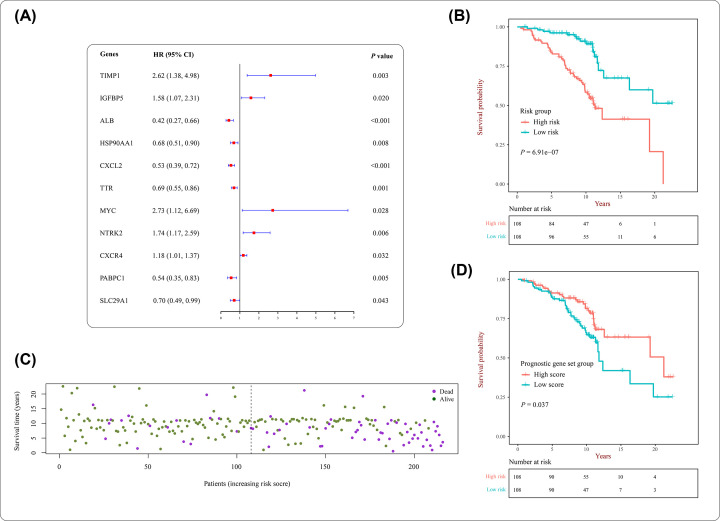
Prognostic analysis of intersecting genes of CHSGS against liver fibrosis (**A**) The results of univariate Cox analysis in patients with liver cirrhosis. (**B**) Survival analysis of the mortality risk scores predicted by 7-gene multivariate cox model. (**C**) Survival status of patients with liver cirrhosis in the high- and low-risk groups. (**D**) Survival analysis of the prognostic gene signature identified by ssGSEA-Cox method.

In order to evaluate whether the 71 intersection genes of CHSGS and liver fibrosis were associated with survival of liver cirrhosis patients, Cox regression, and ssGSEA-Cox method were performed with a bulk RNA-seq dataset of 216 liver cirrhosis patients. After removing the duplicate genes of the bulk RNA-seq data, 18,399 genes expression were retained. Further, except IGHG1, EZR, SGK1, NFKBIA, CXCL8, ATP5F1B, CTSL, and MT-ND4, 63 intersection genes were identified from the comparison of 71 overlapping genes and 18,399 genes of bulk RNA-seq data. Then, univariate Cox analysis of the 63 intersection genes revealed that the survival of liver cirrhosis was significantly associated with 11 genes expression (*P*<0.05), including TIMP1, IGFBP5, ALB, HSP90AA1, CXCL2, TTR, MYC, NTRK2, CXCR4, PABPC1, and SLC29A1 (Figure 8A). However, the multivariate Cox analysis with stepwise regression identified 7 genes out of 11, including SLC29A1, PABPC1, CXCR4, HSP90AA1, ALB, IGFBP5, and TIMP1. Next, the mortality risk scores of patients with liver cirrhosis were predicted through this 7-gene multivariate cox model and the patients were then divided into two subgroups (high- and low-risk subgroups) based on the median value of the mortality risk (Table 1). In the overall survival analysis, the survival rate of high-risk group was significantly lower than low-risk group (*P*<0.001) (Figure 8B). Similarly, the number of deaths increased with the value of risk score (Figure 8C). Moreover, except PRF1, the ssGSEA-Cox analysis identified an optimal prognostic gene signature with 62 genes out of 63 intersection genes. The Kaplan–Meier method with a log-rank test showed that the survival curve of high-enrichment score group was significantly higher than low-enrichment score group (*P*=0.037; Figure 8D).

**Table 1:** Multivariate Cox model of 7-gene in patients with liver cirrhosis

Genes	HR (95% CI)	Risk score (sd)	*P* value
SLC29A1	0.709 (0.462–1.087)	9.581 (0.763)	0.115
PABPC1	0.417 (0.251–0.694)	9.225 (0.644)	0.001
CXCR4	1.139 (0.980–1.324)	9.510 (1.747)	0.090
HSP90AA1	0.629 (0.428–0.926)	11.554 (0.644)	0.019
ALB	0.368 (0.232–0.585)	12.986 (0.402)	<0.001
IGFBP5	2.143 (1.418–3.239)	7.784 (0.641)	<0.001
TIMP1	3.117 (1.496–6.496)	10.587 (0.425)	0.002

sd: standard deviation.

### Docking of core ingredients to core target genes

**Figure 9:**
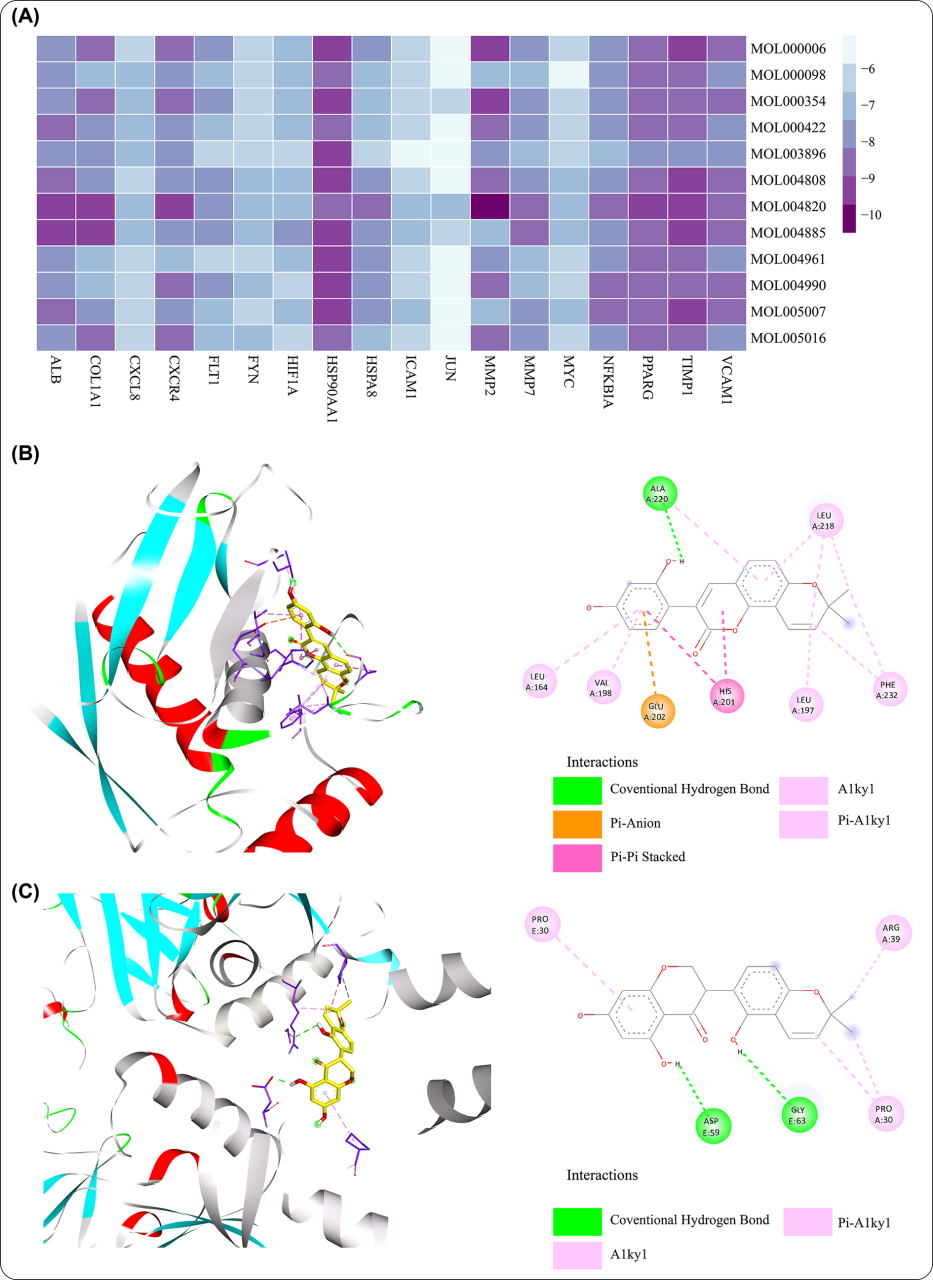
Molecular docking analysis of 12 core ingredients and 18 core target proteins (**A**) The lowest binding energy of 12 core ingredients and 18 core proteins. (**B**) Binding patterns of kanzonols W to MMP2. (**C**) Binding patterns of licoisoflavanone to COL1A1.

In order to analyze the potential binding of core ingredients and core targets in CHSGS against liver fibrosis, molecular docking analysis was performed with MGLTools of Autodock Vina. Except SPP1, IRF1 and CAV1 without the 3D protein structure of Homo sapiens, the rest 18 core target protein structures were obtained from the PDB database and the PDB identifiers were shown in Supplementary Table S7. The molecule structures of 12 core ingredients were then collected from PubChem database. Further, the lowest binding energy of these small molecules and core proteins were calculated by Autodock Vina software. All the absolute value of the lowest binding energy of small molecule-protein were lower than −5 Kcal/mol, indicating that the core target and core pharmacological molecules have strong binding affinity (Figure 9A and Supplementary Table S8). Part of the molecular docking patterns were shown in Figure 9B,C, there were various intermolecular forces such as hydrogen bonds and aromatic bonds between the core molecules and core target protein residues.

## Discussion

In the absence of effective therapies, liver fibrosis progression ultimately leads to cirrhosis. Liver cirrhosis, the main cause of liver-related mortality, which is becoming a global health challenge [3,4]. Moreover, current western therapies are confined to removal of the primary causes and there are no approved antifibrotic drugs for liver fibrosis or cirrhosis. Hopefully, TCM has a natural advantage in treating complex diseases via the interaction network of multiple ingredients and targets. In our previous observational study, CHSGS showed a significant effect on improving the survival of patients with liver cirrhosis [15]. Therefore, we hypothesized that CHSGS may exert an antifibrosis effect through a complex network of multiple ingredients and targets, thereby improving survival of patients with liver cirrhosis.

In the network pharmacology analysis, we first screened out 134 bioactive ingredients of CHSGS through the mentioned ADME models. Based on the target predicting, a total of 1150 target genes of the bioactive ingredients were obtained. Many predicted targets such as TIMP1, MMP2, COL1A1, MMP7, and COL3A1 have been involved in liver fibrogenesis and could be potential therapeutic targets for liver fibrosis [2,7,30]. Among these bioactive ingredients, quercetin (MOL000098), kaempferol (MOL000422), and luteolin (MOL000006) not only had higher number of target genes but were identified as core ingredients of CHSGS against liver fibrosis. Animal experiments have shown that quercetin can ameliorate liver fibrosis by inhibiting HSCs activation and ECM formation [30] and regulating COL1A1, COL3A1, MMP9, and TIMP1 mRNA expression levels [2,30,31]. Besides, it was reported that the natural product kaempferol attenuates hepatic fibrosis by reducing collagen synthesis, suppressing HSCs activation and inhibiting Smad2/3 phosphorylation [32]. Moreover, the antifibrogenic effects of luteolin have been demonstrated by inhibiting proliferation and activation of HSCs and targeting TGFβ/Smad signaling pathway [33].

In order to identify liver fibrosis-associated genes, the DEG analysis was performed with scRNA-seq data of hepatic NPCs. Liver fibrosis involves complex interactions between multilineages of NPC located within fibrous scar regions, known as fibrotic niche [17]. In response to liver injury, these NPCs interact to promote fibrosis via multiple signaling pathways, such as PDGFR, NOTCH, TNFRSF12A, and TGF-β1/Smad2/3 pathways [17,32]. HSCs are not only an important lineage of NPCs but also play a key role in liver fibrogenesis. When stimulated with fibrogenic cytokines, quiescent HSCs turn into activated HSCs, secreting ECM to form fibrous scar regions [2]. Application of scRNA-seq method, TREM2^+^CD9^+^ macrophages and ACKR1^+^PLVAP^+^ endothelial cells were identified and expanded in liver fibrosis [17]. Besides, MDMs and Kupffer cells can produce TGFβ and that is the key mediator to the liver fibrogenesis [34,35]. NK cells are also involved in the pathogenesis of liver fibrosis [36], the relative absence of CD56^+^ and CD16^−^ NK cells subpopulation in cirrhotic liver was found with scRNA-seq [17]. Previous mouse models indicated that liver B cell has a role in regulating liver fibrosis [37,38]. Compared with the bulk RNA-sequencing, scRNA-seq technology can analyze transcriptome at a single-cell resolution that leads to novel insights into disease mechanisms [39,40]. Furthermore, we can accurately identify target genes from the NPCs through analyzing the scRNA-seq data, while the bulk RNA-seq obtains target genes from bulk cell populations including NPCs and other cell lineages in liver. Thus, the NPCs population was identified from the two combined scRNA-seq data. With the subsequent DEG analysis, 625 fibrosis-associated genes were retained.

Following 71 intersection genes of CHSGS against liver fibrosis were identified from the scRNA-seq data analysis of liver NPCs and target predicting of CHSGS, of which 25 were up-regulated and 46 were down-regulated. In order to determine whether the intersection genes were associated with the survival of liver cirrhosis patients, 63 intersection genes were used to perform Cox regression and ssGSEA-Cox method. First, univariate Cox analysis determined 11 genes that were significantly associated with survival of liver cirrhosis. Further, a 7-gene multivariate cox model was performed to predict the mortality risk score of patients with liver cirrhosis. Next, based on the median value of the mortality risk, the survival rate of high-risk group was significantly lower than low-risk group. It indicated that CHSGS may improve survival of patients with liver cirrhosis through the interaction of seven genes. Moreover, considering that TCM treats liver cirrhosis through the complex network of multiple ingredients and targets, we used the ideal of gene enrichment approach to determine the optimal prognostic gene set of CHSGS against liver cirrhosis. The ssGSEA method, an extension of GSEA, can calculate the enrichment scores of the given gene set in each sample [28]. Meanwhile, the Cox analysis was used to correlate survival with enrichment risk scores. Final, ssGSEA-Cox analysis identified an optimal prognostic gene signature with 62 intersection genes, which was significantly associated with the survival of liver cirrhosis. The 62-gene signature reflected that CHSGS has characteristic of treating cirrhosis through multiple targets. Further, it can be inferred that CHSGS may prolong survival by the prognostic gene signature, which may be used for determining survival in cirrhotic patients.

With the network centrality method of identifying core genes, 21 core genes were identified, of which 8 were up-regulated and 13 were down-regulated. Among the up-regulated genes, TIMP1, COL1A1, and SPP1 showed a higher level of log fold change. As is known to all, TIMP1 (tissue metalloproteinase inhibitor 1) can inhibit vast majority of matrix metalloproteinases, which is the only enzyme that can break down the fibrous collagen [41]. COL1A1 (type I collagen), as the mainly ECM collagen, have a critical role in liver fibrosis [7]. Previous study also showed osteopontin encoded by SPP1 can enhance and promote liver fibrosis [42]. Enrichment analyses of these core genes showed that cellular response to external stimulus, oxidative stress, inflammation response, ECM remodeling, NOS3 activation and regulation and VEGF signaling pathway were the predominant biological processes and pathways in CHSGS treating liver fibrosis. Notably, 5 out of 8 core up-regulated genes were involved in ECM degradation, collagen degradation, collagen metabolic process and activation of matrix metalloproteinases, indicating CHSGS could have the antifibrotic effects of promoting fibrosis resolution. Besides, regression of hepatic fibrosis is accompanied with a reduction of pro-inflammatory cytokines such as IL-17, and IL-1β in the liver [2]. Meanwhile, both up-regulated and down-regulated genes were related to interleukin-associated signal pathway, suggesting CHSGS may regulate the release of inflammatory cytokines. NOS3 gene, encoding nitric oxide (NO) synthase in endothelial cells, produces NO that can regulate VEGF (vascular endothelial growth factor)-induced angiogenesis [43]. In the meantime, research has shown that procyanidin B2 can inhibit the proliferation of HSCs and angiogenesis by the hedgehog pathway and down-regulation of VEGF-A expression [44]. In summary, these data revealed that CHSGS may exert antifibrosis effects by modulating inflammation response, inhibiting angiogenesis and regulating ECM remodeling, especially in ECM remodeling. Molecular docking analysis found that the above core genes and ingredients had strong binding affinity, which further illustrated the important roles of core genes/ingredients in the treatment of liver fibrosis.

## Conclusions

Taken together, the multi-omics and network pharmacology findings highlight that the key mechanisms of CHSGS against liver fibrosis include modulation of inflammation responses, inhibition of angiogenesis, and regulation of ECM remodeling, of which the most important mechanism was the regulation of ECM remodeling. In addition, 62-gene signature may be used for determining the prognosis in cirrhotic patients based on the results of ssGSEA-Cox analysis. Moreover, the potential pharmacological ingredients and targets of CHSGS against liver fibrosis were identified, indicating that CHSGS may serve as an effective antifibrotic therapy.

## Supplementary Material

Supplementary Tables S1-S8

## Data Availability

The data presented in this study are available in this article (summarized in figures and tables).

## Abbreviations

CHSGS: Chaihu-Shugan-San
H-I-T-D: herb-ingredient-target-disease
HSC: hepatic stellate cell
LSEC: liver sinusoidal endothelial cell
MDM: monocyte-derived macrophage
NPC: nonparenchymal cell
PPI: protein–protein interaction
VEC: vascular endothelial cell
